# Using the eServices Platform for Detecting Behavior Patterns Deviation in the Elderly Assisted Living: A Case Study

**DOI:** 10.1155/2015/530828

**Published:** 2015-03-22

**Authors:** Isabel Marcelino, David Lopes, Michael Reis, Fernando Silva, Rosalía Laza, António Pereira

**Affiliations:** ^1^Higher Technical School of Computer Engineering, University of Vigo, Polytechnic Building, Campus Universitario As Lagoas s/n, 32004 Ourense, Spain; ^2^INOV INESC Innovation, Institute of New Technologies of Leiria, 2411-901 Leiria, Portugal; ^3^School of Technology and Management, Computer Science and Communications Research Centre, Polytechnic Institute of Leiria, 2411-901 Leiria, Portugal

## Abstract

World's aging population is rising and the elderly are increasingly isolated socially and geographically. As a consequence, in many situations, they need assistance that is not granted in time. In this paper, we present a solution that follows the CRISP-DM methodology to detect the elderly's behavior pattern deviations that may indicate possible risk situations. To obtain these patterns, many variables are aggregated to ensure the alert system reliability and minimize eventual false positive alert situations. These variables comprehend information provided by body area network (BAN), by environment sensors, and also by the elderly's interaction in a service provider platform, called eServices—Elderly Support Service Platform. eServices is a scalable platform aggregating a service ecosystem developed specially for elderly people. This pattern recognition will further activate the adequate response. With the system evolution, it will learn to predict potential danger situations for a specified user, acting preventively and ensuring the elderly's safety and well-being. As the eServices platform is still in development, synthetic data, based on real data sample and empiric knowledge, is being used to populate the initial dataset. The presented work is a proof of concept of knowledge extraction using the eServices platform information. Regardless of not using real data, this work proves to be an asset, achieving a good performance in preventing alert situations.

## 1. Introduction

Currently modern society's members are facing a fast paced lifestyle. People are overloaded. Meanwhile life expectancy is increasing [1]. As a consequence, the less elderly have proper care and attention from their family members. This lack of care may result in several issues, since senior population is minded to have a more fragile health and physical condition. In a physiological side, the elderly are more likely to develop loneliness and insecure feelings if they are alone in their own homes or might feel a burden, outsider, and losing their autonomy when admitted to long term care facility. The elderly's family members can also put forth concern and guilty consciousness for not having time to be present for their seniors. In a financial side, the cost of being in a nursing home might be unbearable to the senior, the family, or the government [2].

Having this motivation in mind, our research team has developed a service platform with services targeted to the senior population, called Elderly Support Service Platform (eServices). It combines the concern towards physical aspects, as well as social and physiological aspects.

eServices provides continuous monitoring of vital parameters and a person location in their natural environment by wireless and noninvasive technologies. This permanent monitoring associated with distress triggers ensures that the elderly have an immediate response in eminent danger situations. It also potentiates building a large dataset that helps making more accurate diagnosis, as doctors have substantially more data to base their decisions. All of the collected information can also enable a preventive response along with the reactive response, since deviations may be detected and treated earlier. Additionally, as patient records are digital and available through networks, all stakeholders (doctors, nurses, firefighters, etc.) are able to quickly access relevant information whenever it is necessary (if they have the permissions to do so).

As previously mentioned, eServices also includes features to overcome social and physiological issues. The elderly have, in their natural environment, a device where they can easily access a wide range of scalable services that meet their needs and expectations (medical services, maintenance services, leisure services, cultural services, experience record services, and call center). Among other things, they can have virtual doctor appointments; play card games with their social network friends; or record their experiences to share with others.

All of the data used in eServices (biosensors, environment sensors, and the log resultant from the interaction between the services and the elderly) can be analyzed, allowing the detection of possible risk situations.

These data are only translated into knowledge if data mining techniques are applied. It is almost impossible for a human being to detect and extract information in such large datasets. When monitoring a person's behavior does detect potential risk situations, it is also important to be aware of false positives and false negatives. False positives can jeopardize the alert system's credibility whenever a distress flag is raised in a situation where nothing has really happened. False negatives are even more harmful and can put a life at risk due to the fact of missing the detection of a real alarm situation.

This paper presents the knowledge extraction procedure from the eServices dataset using Cross-Industry Standard Process for Data Mining (CRISP-DM) [3] methodology and emphasizes a solution that narrows down false negatives. Decision trees, built with the C5.0 algorithm, are used as a predictive data mining modeling method.

Since the eServices platform is still under development and in testing, there are not yet sufficient quality data to proceed with proper knowledge extraction. With that in mind, we have derived a dataset based on the existing data and empirical awareness. This dataset has origins in a sample of data obtained by eServices sensors, in meteorology records, and in the demographic data of its users. Then, a set of rules was inducted in the dataset in order to later try to find them through the CRISP-DM process, as we describe in Section 4. Although we are aware that the conclusions regarding the resulting rules are not valid for real world appliance and that this process will necessary be repeated and revised when working with real data, this work intends to be a proof of concept on how pattern deviations may be detected using the eServices platform.

The remainder of the paper proceeds as follows: related work is presented in Section 2, followed by the summarization of eServices platform in Section 3. Section 4 approaches the core of this paper concerning knowledge extraction to unequivocally identify potential risk situations. Conclusions and future work are presented in Section 5.

## 2. Related Work

Knowledge extraction and its use to predict future situations have been a challenge applied to several areas, including health. The existence of large datasets of information led to the need of developing technologies that are able to quickly analyze them. Online analytical processing (OLAP) technology, data warehouse (DW), knowledge discovery in databases (KDD), and data mining (DM) are some of the techniques that, when combined, allows suppressing the mentioned requisites.

A DW is a database separated from the different data sources where the data are clean, uniformed, and consolidated. Firstly, data sources are identified, then the extract, transform, and load (ETL) process is made. Afterwards, OLAP enables DW to provide rapid responses to iterative complex analytical queries. OLAP cubes, or more recently Microsoft tabular model, feed the presentation layer providing ad hoc and static reporting, dashboards, scorecards, and key performance indicators (KPI) to help in the decision making.

**Figure 1:**
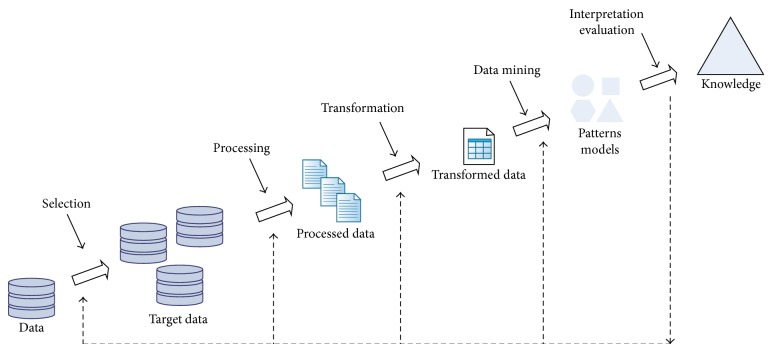
KDD process (adapted from [3]).

KDD is a process to discover hidden knowledge from massive amounts of data, as Figure 1 illustrates. It starts with data selection and data preparation through cleansing and transformation processes. Afterwards, DM is applied to find patterns which are later interpreted and evaluated [4]. DM can be defined as “the process of discovering meaningful new correlations, patterns, and trends by sifting through large amounts of data stored in repositories, using pattern recognition technologies as well as statistical and mathematical techniques” [5].

There are many literature reviews that enhance the importance of using these concepts and technologies. In [6] a literature review is made to show the DM techniques trends in the past decade.

Concerning the health field, [7] presents a literature review with 24 solutions in medical DM, indicating techniques, utility, and diseases. Another literature survey of DM in healthcare and biomedicine is made by [8]. Other contributions in this field are made by [9, 10] where a wireless sensor network (WSN) that detects the elderly's basic daily routines in order to predict senior's behavior is presented. A home based health monitoring to mine human patterns is also presented in [11].

Regarding the papers that present literature reviews, many of them make an analysis on how to overcome issues related with the DM process. One of the issues pointed out by [8] when using DM in healthcare is the low quality of patient data. Another problem highlighted by [12] is the difficulty of handling heterogeneous data sources like laboratory results, doctors' appointments, and also the absence of DW to consolidate data. The lack of treated data to feed the DM process is also remarked by [13]. It is also clear that the information that is mostly analyzed to predict problems in senior population is based on basic life signs (like blood pressure, temperature) or routines achieved through environment sensors.

In order to suppress the above-mentioned issues, we have developed a solution that gathers and combines information from the basic life signs and environment sensors and from the elderly's interaction with the eServices platform. By aggregating all of these data we are able to predict more accurately distress situations. Moreover, the use of an ETL process to extract all of the information from our several data sources, transform it, clean it, and consolidate it in order to load it into a DW reduces the issues that may appear in DM. Additionally, having a platform that enables adding services in a scalable mode will increase the amount of information collected and, therefore, the distress situation predictively. This central and integrated data repository empowers the DM process to easily obtain the necessary data.

In the next section, the eServices platform is summarily presented to illustrate the information that it can provide.

## 3. Elderly Support Cloud Service Platform

The main purpose of the eServices platform is to aggregate services targeted to the elderly population. The major concern on its conception was to create an easy to use and simple interaction system due to the elderly's special needs. Another relevant preoccupation was to include physical health issues, but also to act in a social and psychological aspect [14–16].

**Figure 2:**
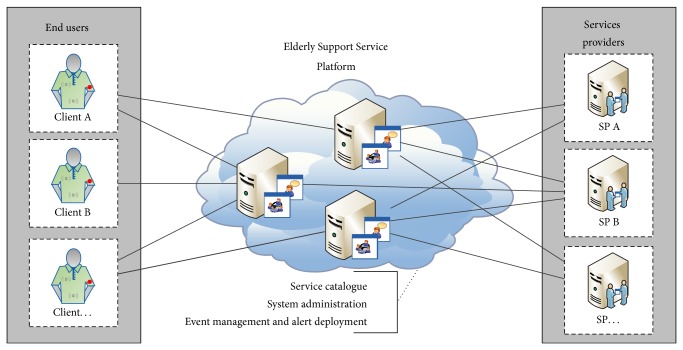
eServices—Elderly Support Service Platform.

Concerning eServices architecture, there are three major segments: end user clients that consume services, service provider that supplies services, and cloud service provider platform, as shown in Figure 2.

Regarding end user clients, users will have biosensors to monitor basic life signs as well as environment sensors to obtain surrounding information and a device to interact with some of the available services.

As for service providers, they supply services in a specific category in the enterprise service bus (ESB), according to an established protocol.

The cloud service provider platform will not only provide a wide range of services but also have system administration and event management and alert deployment features. System administration attends issues such as authentication, authorization, access control, and security. Since sensitive information is gathered (including vital parameters, medical history, and daily routines) quasi-identifiers are avoided and user identities are protected from service providers. Event management and alert deployment are responsible for analyzing the obtained information, perceive threat situations, and trigger the adequate response. The present paper is focused on the event management.

Therefore, eServices encompasses monitoring basic life signs through BAN and offers a large range of services that can currently be categorized by medical services, maintenance services, leisure services, cultural services, experience record services, and call center. The mentioned categories can be extended and the services provided in each category are scalable. To remove any installation and configuration issues, all of the services are available as software as a service (SaaS), nested on the cloud.

BAN monitoring and information acquisition are being presented by our research team in [17–20]. A special attention is being paid to fall detection since it is one of the most important issues in detecting danger situations.

Regarding leisure services, several games that the elderly usually like are available, as well as games to promote mental stimulation. Other games might be added with the use of tools such as Microsoft Kinect or Wii fit, to detect and correct possible posture deviations as well as to endorse physical exercise, maintaining the elderly's health. Also in leisure services, our research team has implemented an interactive musical environment for gerontechnology [21].

As for medical services, virtual appointments can be done to complement diagnoses in situations where seniors are unable to address a health area (hospital or others). This kind of service, without replacing face-to-face consults, allows narrowing links between seniors and health carriers (doctors, nurses, caregivers, and others) by having a simple and low cost efficient way to communicate.

In experience record services, the elderly may record all of their tacit knowledge and share it with other seniors or family members. The idea is to avoid losing empiric knowledge in areas like agriculture, handicraft, and cooking, allowing this information to pass from generation to generation.

Many other services are already in use in the eServices platform, seeking to improve the elderly's quality of life by maintaining them as part of the active population and as contributors to society knowledge. These services also increase secure and safety feelings due to continuous monitoring and proper response in danger situations.

In the next section the methodology to identify alert situations is presented.

## 4. Detecting Risk Situations

CRISP-DM is based on KDD and is the most widely used methodology for developing data mining projects [22]. This methodology is applied in the present study.

**Figure 3:**
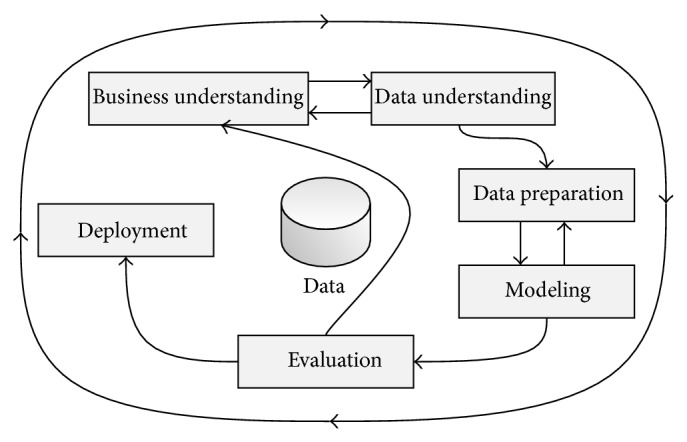
CRISP-DM life cycle [7].

The life cycle of CRISP-DM includes six phases: business understanding, data understanding, data preparation, modeling, evaluation, and deployment (Figure 3).

The first phase is to understand the business process, clarify the concepts, and realize what results are being expected (business understanding). The second phase includes analyzing and exploring the available data and understanding what it means (data understanding). Then data should be prepared, outliers must be treated accordingly, new data may be created by aggregations, data can be formatted, and so forth. These operations intend to simplify data to be processed by DM's algorithms, constructing the final dataset (data preparation). Afterwards models are built from DM's algorithms (modeling) [23].

DM's algorithms are classified into two categories: descriptive or unsupervised learning and predictive or supervised learning. In descriptive data mining, clusters are created by aggregating similar records to discover unknown patterns or relations between data. It includes clustering, association rules, sequence discovery, and summarization tasks. In predictive mining, prediction rules are applied to training data and further applied to unclassified data. It includes classification, regression, time series analysis, and prediction [8].

Evaluation is the phase where the prediction quality of the models built is evaluated (evaluation). In a satisfactory evaluation, the solution is deployed to production mode (deployment). Otherwise the process is made again. Nevertheless, even when good results are obtained, all the process is occasionally redone to detect new rules or trends, in order to monitor eventual changes.

The data sources in the eServices platform are the inputs obtained from the interaction of the elderly with the services present in the platform, data provided by biosensors along with environment sensors. Other relevant inputs might be added. The conjunction of all of the information feeding our system intends to infer deviations that may indicate alert situations.

Since the platform is in development, synthetic data were used to validate the DM process. Some parameters were considered based on an existing sample and an empirical evaluation: the existence of 50 users, 1 year of platform use, 3 profiles (high usage, average usage, and low usage), 75 years old as average with 10 years of standard deviation, and marital status according to the following distribution: 30% married, 50% widowed, 10% single, 8% divorced, and 2% cohabitation.

The initial dataset was constructed with 19 attributes that characterize access for a 3-day time window for each user.

In order to facilitate the DM process evaluation, alerts were generated based on well-defined rules.

*1st Rule*. If the user does not access the platform during 3 consecutive days and none of them are weekends or holidays and the user belongs to the high or average use profile then alert. 

*2nd Rule*. If the user's age is between 70 and 80 inclusive and their marital status is divorced, widowed, or single and the access occurs between 11 p.m. and 7 a.m. and its duration is over 3 hours then alert. 

*3rd Rule*. If the user's age is above 70 and their marital status is married and the access is over 3 hours then alert. 

*4th Rule*. If the temperature is between 15 and 30°C and the atmospheric condition is sky clear, partly cloudy, or with scattered clouds and the access is over 3 hours then alert. 

*5th Rule*. If the user accesses the platform 3 consecutive weekends and misses the next then alert.

During the dataset construction some random errors were generated in order to provide a more realistic scenario that further trigger issues to be solved in data preparation.

The following subsections present the CRISP-DM methodology applied to our problem.

### 4.1. Data Understanding

The first step was to target data sources, understand its meaning, and identify necessary data transformation. Afterwards, the data stating area (DSA) structures and data marts were created. Finally the ETL process was made in order to populate the DW. The ETL process and the DW construction allow us to treat information and suppress some data preparation made in DM.

The original dataset was made based on the information contained in the DW and comprises 19 attributes. The list presented below provides information on each one of these attributes and information about their meaning:

- Surrogate_Key: user identification in the DW;
- Age: user age;
- District: user's residence district;
- Marital_Status: user's marital status;
- Alert_Date: date of occurrence of an alert situation;
- Num_Accesses: number of accesses (distinct sessions) that the user has made in the eServices platform;
- Num_Accesses_Holidays_Weekends: number of accesses that the user has done to the eServices platform in holidays and weekends;
- Num_Accesses_Morning: number of accesses that the user made to the eServices platform during mornings (7:00–12:00);
- Time_Session_Max: longest access to the eServices platform, in minutes;
- Time_Session_Min: smallest access to the eServices platform, in minutes;
- Time_Offline_Max: longest time offline, in minutes;
- Time_Offline_Min: smallest time offline, in minutes;
- Temperature_Max: maximum temperature value in the region where the user accessed the eServices platform;
- Temperature_Min: same as Temperature_Max, but for minimum temperature;
- Humidity_Max: maximum humidity value in the region where the user accessed the eServices;
- Humidity_Min: same as Humidity_Max, but for minimum temperature;
- Is_Alert: flag that indicates if there was an alert in a 3-day window before each access.

### 4.2. Data Preparation

Despite the cleansing process made in the ETL process, it was necessary to make some additional transformations such as outlier detection, attribute discretization, and attribute removal or addition in order to prepare data for further analysis and classification. The inclusion of irrelevant, redundant, and noisy attributes in the DM process can result in hard knowledge discovery [24]. Data exploration allowed us to perceive irregularities to treat. In data exploration, statistics regarding unique values, value ranges, maximum and minimum value, and average and missing values were made for each attribute.

As a result, we have observed some outlier values in temperature and humidity such as −9999 and 100. We have decided to replace those values with blank. Another possible approach was to replace them by average or mode values of the attribute, but in this case the usage of average or mode values can jeopardize the collected sample by introducing incorrect numbers.

Concerning attribute discretization and attribute removal or addition, the list bellow shows, for each attribute, the actions taken:

(i) Surrogate_Key: since we want to infer generic rules there is no relevancy in keeping the DW identifier. Action: attribute removal;
(ii) Age: age attribute is very important for our analysis and must be kept. Nevertheless, there are innumerous different values. Action: set value ranges;
(iii) District: it does not seem relevant to achieve user's district as it is possible to access the eServices platform from different locations. Action: attribute removal;
(iv) Marital_Status: user marital status can have a great influence in its habits; therefore it is an attribute to keep. For instance, singles or widows may feel more solitude and spend more time online. Action: keep attribute;
(v) Alert_Date: this attribute is essential in the data preparation phase in order to aggregate different information. However it has so many distinct values that it will not offer any additional relevant information to DM algorithms. Action: attribute removal;
(vi) Num_Accesses: it may be a relevant attribute, so it is maintained. Action: keep attribute;
(vii) Num_Accesses_Holidays_Weekends: Holidays and weekends can have an impact on user's behavior, as they potentially have diverse routines like sightseeing or visiting family. Therefore, the attribute is kept. Action: keep attribute;
(viii) Num_Accesses_Morning: it can be interesting to cross this information with the Num_Accesses_Holidays_Weekends attribute. Action: keep attribute;
(ix) Time_Session_Max: it seems relevant to obtain information about different access during day periods of time. Action: keep attribute;
(x) Time_Session_Min, Time_Offline_Max, Time_Offline_Min: it may be a relevant attribute, so it is maintained. Action: keep attribute;
(xi) Temperature_Max, Temperature_Min, Humidity_Max, Humidity_Min: the weather can influence how users act. Temperature in conjunction with humidity can provide relevant atmospheric conditions. Nice weather may lead seniors to spend more time outdoors. Action: keep attribute;
(xii) Is_Alert: this attribute is the DM process goal characteristic. Action: keep attribute.

Also, in data preparation CRISP-DM phase it is possible to derive new attributes that best represent the existing information or to simplify DM algorithms processing. This derivation can result from existing attributes aggregation, or by applying other DM algorithms, or as a result of attribute value discretization.

As previously mentioned, separate attributes like temperature (*t*) and humidity (*h*) might not give important information. However atmospheric conditions (ac) may be obtained by combining these two attributes results (rt, rh):

 
*t* < 15 → rt = 0;
 15 < *t* < 30 → rt = 1;
 
*t* > 30 → rt = 2;
 
*h* = “Rain”∣“Light  Rain  Showers” → rh = 0;
 
*h* = “Partly  Cloudy”∣“Scattered  Clouds”∣*“*Clear” → rh = 2;
 
*h* = Other  situations → rh = 1;
 ac = rt + rh.

Atmospheric conditions (ac) were added as a new attribute in the dataset.

Another derived attribute is profile that represents user's profile based on cluster algorithms. For this purpose we have used the two-step cluster algorithm. Observing the offline time in minutes, we were able to group data into three clusters: high usage, average usage, and low usage.

The two-step cluster algorithm was also applied to age attribute. We have observed that there were many distinct values in this attribute and that there was the need to discretize them. After observing the clusters formed by the algorithms, we have decided to empirically give more meaning to this range of values:

 two-step cluster [−*∞*, 50], ]50, 60], ]60, 70], ]70, 80], ]80, 90], ]90, 100], ]100, +*∞*];
 empirical ranges [−*∞*, 65], ]65, 80], ]80, 90], ]90, +*∞*].

The same approach was made to session times. Regarding the session time, the two-step cluster pointed out 25 minutes; however we consider that it is more appropriate the discretization of this attribute in 30-minute ranges. As for the offline time, the algorithm suggests an 8-hour division, but we have opted for 12-hour clusters.

Finally, we have perceived that most of the data are in a format that is easily identified by DM algorithms. The only data conversion made was in the Is_Alert that has the values “yes” and “no” in the DW and was converted to 1 and 0 and also in the Marital_Status that were passed from a numeric value to a string value.

As a result of the data preparation, the final dataset to provide to DM algorithms was compiled.

### 4.3. Modeling

DM's purpose is to extrapolate rules from the data existing in the dataset. The choice of the algorithm depends on the problem to solve, the gathered data, and the computing tools available. For our problem we have used C5.0 decision tree learning algorithm since they are easily understood and interpreted [25].

To evaluate the model's predictive ability, the model must be trained with a known dataset and validated with an unknown dataset. The ideal model would have 100% hit rate, a receiver operating characteristic (ROC) of 1, based on the relation between true and false positives, no false positives or false negatives.

In the present context, models that enhance less false negatives are going to be overrated over models that value false positives. It is preferable that the system throws an alert when nothing happens to the service user compared to missing an alert in a distress situation.

Record partition for training and validation was based on 10-fold cross-validation. This process involves randomly dividing the data into 10 equal size subsamples. A model is built and evaluated in each iteration. After all of the iterations a new model is built with all of the training dataset to ensure that the model was built with the most of the information available. The average metrics obtained in each of the 10 iterations were the evaluation metrics considered.

In the model construction using C5.0 no limits were introduced in maximum purity, maximum depth, and minimum number of records per leaf nodes parameters, to avoid narrowing down the obtained rules.

Two approaches were made to create the models. In the first approach, all of the records were supplied to the DM algorithm. In the second approach three distinct models were created, each one for a specific profile previously detected by the clustering algorithm. For both approaches, several models were generated: some where no cost matrix is provided and others where it is.

Therefore, 8 models were created.  Table 1 shows, for each model, the records considered and the cost matrix application.  Table 2 presents the rules for each model.

### 4.4. Evaluation

Several models with different rules were proposed to predict the alert occurrence. To assess the model's quality and consequently the applied rules, an analysis was made for each model. This analysis was based on hit rates and the area under the ROC curve.  Table 3 shows the obtained values for each model.

From the values presented in Table 3 it is clear that models 5 and 6 are to rule out, since the area under the ROC curve is very small. Also in models 1 and 2, which are applied to all records, the area under the ROC curve is slightly above 0.5 and the hit rates regarding alerts are very poor. All of the remaining models have relevant values that are useful in the problem resolution.

Afterwards, it is important to evaluate the model across a confusion matrix that represents the number of correct and incorrect predictions. This analysis will also provide information on the influence of the cost matrix application.  Table 4 represents the confusion matrix for the models that were not discarded in the previous step.  Table 5 shows the results obtained by the values in confusion matrix.

The rules from the selected models that classify a record as being an alert are as follows:

 Time_Session_*Max*⁡≥180∧Age > 70 → Is_Alert = true;
 Time_Session_*Max*⁡≥120∧Atmospheric_Conditions ≥ 1.5 → Is_Alert = true.

Testing these rules in the initial dataset, with all of the records, we obtain the confusion matrix shown in Table 6.

Concerning the number of correct predictions, 5152 records are correctly predicted, while 898 records are not correctly classified. This implies a 0.85 accuracy rate and therefore a 0.15 error rate.

### 4.5. Deployment

After obtaining the models it is important to understand how the DM process will continue in the future.

In this situation, the test data will be removed from the DW and the ETL process will be scheduled to continuously feed the DW. The DM process must be redone with some frequency to determine whether the found rules are still valid or if new rules are detected.

## 5. Conclusions and Future Work

Concerning our major goal to detect behavior deviations in senior population it is imperative to combine several data in order to be as accurate as possible. Using eServices platform, both physical parameters (basic life signs and environment) and the elderly's interaction with the platform are being assembled in a large dataset, which can be enriched by adding more services.

After briefly presenting the eServices platform where the data are achieved, we have focused on the use of the CRISP-DM methodology to obtain a valid model that is able to predict alert situations based on the gathered data. Since the platform is still in development, synthetic data was used to validate the DM process based on a real data sample obtained by eServices sensors, meteorological data, and empiric domain knowledge. During this process we have implemented a DW. As previously mentioned, the presence of an ETL process and the data consolidation using a DW facilitates the DM work, as many of the data treatments are made by the ETL process. The existence of the DW will also allow assisting decision support systems through reporting, KPI, dashboards, and scorecards.

Although our solution makes use of a DW that allows obtaining a set of rules through the CRISP-DM process, helping on the detection of possible pattern deviation behaviors, there are real life situations where time is critical and it is necessary to act immediately according to the information provided by the BAN. This means that many alert situations must be triggered without waiting for the ETL process to occur. This immediate response is, in our system, granted by the BAN. In the DW we will further register if the triggered alert was indeed a valid distress.

Subsequently, it is vital to explore data, manage to remove any noise and redundant information, compile it, and proceed with several operations to promote an easy knowledge discovery from DM algorithms. We have used C5.0 and generated several models. From the 8 models created and evaluated, the largest contribution for solving the problem obtains a 0.85 accuracy rate. These results were also confirmed by the confusion matrix.

Summarily, in this paper, a proof of concept is presented showing the KDD process made to detect risk situations in eServices platform. The presented work allowed us to explore a useful methodology to apply as future guidance. It also allowed us to build a scenario where the information is extracted from the inputs of the eServices platform into a DW leading to consolidated data. Regarding future work, as data are being collected in senior's homes, the proof of concept will be applied using real data.

## Figures and Tables

**Table 1:** Models.

Model	Profile	Cost matrix application
M1	All records	No
M2	All records	Yes
M3	Low usage profile	No
M4	Low usage profile	Yes
M5	Average usage profile	No
M6	Average usage profile	Yes
M7	High usage profile	No
M8	High usage profile	Yes

**Table 2:** Model's rules.

Model	Rules	
M1	R1	*Time*_*Session*_*Max* ≥ 180∧*Profile* < 1,5∧*Age* ≥ 70 → *Is*_*Alert* = *true*
	R2	*Time*_*Session*_*Max* ≥ 180∧*Profile* < 1,5∧*Age* < 70 → *Is*_*Alert* = *false*
	R3	*Time*_*Session*_*Max* ≥ 180∧*Profile* ≥ 1,5∧*Age* ≥ 70 → *Is*_*Alert* = *false*
	R4	*Time*_*Session*_*Max* < 180∧*Atmospheric*_*Conditions* < 1,5 → *Is*_*Alert* = *false*
	R5	*Time*_*Session*_*Max* < 180∧*Atmospheric*_*Conditions* ≥ 1,5 → *Is*_*Alert* = *true*
	R6	120 ≤ *Time*_*Session*_*Max* < 180∧*Atmospheric*_*Conditions* ≥ 1,5 → *Is*_*Alert* = *false*

M2	R1	*Time*_*Session*_*Max* < 180 → *Is*_*Alert* = *false*
	R2	*Time*_*Session*_*Max* ≥ 180∧*Profile* < 1 → *Is*_*Alert* = *false*
	R3	*Time*_*Session*_*Max* ≥ 180∧*Profile* = 1∧*Age* < 70 → *Is*_*Alert* = *false*
	R4	*Time*_*Session*_*Max* ≥ 180∧*Profile* = 1∧*Age* ≥ 70 → *Is*_*Alert* = *true*

M3	R1	*Time*_*Session*_*Max* < 180 → *Is*_*Alert* = *false*
	R2	*Time*_*Session*_*Max* ≥ 180∧*Age* < 70 → *Is*_*Alert* = *false*
	R3	*Time*_*Session*_*Max* ≥ 180∧*Age* ≥ 70 → *Is*_*Alert* = *true*

M4	R1	*Time*_*Session*_*Max* < 180∧*Atmospheric*_*Conditions* < 1,5 → *Is*_*Alert* = *false*
	R2	*Time*_*Session*_*Max* < 180∧*Atmospheric*_*Conditions* ≥ 1,5 → *Is*_*Alert* = *trus*
	R3	*Time*_*Session*_*Max* ≥ 180∧*Age* < 70 → *Is*_*Alert* = *false*
	R4	*Time*_*Session*_*Max* ≥ 180∧*Age* ≥ 70 → *Is*_*Alert* = *true*

M5	R1	*Time*_*Session*_*Max* ≥ 180∧*Atmospheric*_*Conditions* < 1,5∧*Age* ≥ 70∧*Marital*_*Satus* ≠ *Married* → *Is*_*Alert* = *true*
	R2	*Atmospheric*_*Conditions* < 1,5 → *Is*_*Alert* = *false*
	R3	*Time*_*Session*_*Max* < 120∧*Atmospheric*_*Conditions* ≥ 1,5 → *Is*_*Alert* = *false*
	R4	*Time*_*Session*_*Max* ≥ 120∧*Atmospheric*_*Conditions* ≥ 1,5 → *Is*_*Alert* = *true*

M6	R1	*Is*_*Alert* = *true*
	R2	*Atmospheric*_*Conditions* < 1,5 → *Is*_*Alert* = *true*

M7	R1	*Atmospheric*_*Conditions* < 1,5 → *Is*_*Alert* = *false*
	R2	*Time*_*Session*_*Max* ≥ 120∧*Atmospheric*_*Conditions* ≥ 1,5 → *Is*_*Alert* = *true*
	R3	*Time*_*Session*_*Max* < 120∧*Atmospheric*_*Conditions* ≥ 1,5 → *Is*_*Alert* = *false*

M8	R1	*Atmospheric*_*Conditions* < 1,5 → *Is*_*Alert* = *false*
	R2	*Time*_*Session*_*Max* ≥ 120∧*Atmospheric*_*Conditions* ≥ 1,5 → *Is*_*Alert* = *true*
	R3	*Time*_*Session*_*Max* < 120∧*Atmospheric*_*Conditions* ≥ 1,5 → *Is*_*Alert* = *false*

**Table 3:** Model's evaluation.

Model	Hit rate Is_Alert = false	Hit rate Is_Alert = true	Area under the ROC curve
M1	0,98	0,499	0,551
M2	0,98	0,499	0,551
M3	0,858	0,945	0,814
M4	0,85	0,955	0,823
M5	0,983	0,244	0,254
M6	0,983	0,244	0,254
M7	0,989	0,889	0,881
M8	0,969	1	0,986

**Table 4:** Confusion matrix.

	Predicted true	Predicted false
M3		
Actual true	445	26
Actual false	105	634

M4		
Actual true	450	21
Actual false	111	628

M7		
Actual true	24	3
Actual false	19	1769

M8		
Actual true	27	0
Actual false	56	1732

**Table 5:** Evaluation's models results.

Model	Correct predictions	Incorrect predictions	Error rate	Accuracy rate
M3	1079	131	0,11	0,89
M4	1078	132	0,11	0,89
M7	1793	22	0,01	0,99
M8	1759	56	0,03	0,97

**Table 6:** Confusion matrix from initial dataset with final rules.

	Predicted true	Predicted false
Actual true	615	277
Actual false	621	4537
